# Seven days, seven quests: gamification to enhance engagement with antimicrobial stewardship resources during Antibiotic Awareness Week

**DOI:** 10.1017/ash.2026.10320

**Published:** 2026-03-31

**Authors:** Meghan N. Jeffres, Thalia McCann, Madison E. Salam, Karrine D. Brade

**Affiliations:** 1 Skaggs School of Pharmacy and Pharmaceutical Sciences, University of Colorado Anschutz, USA; 2 University of Colorado Hospital: UCHealth University of Colorado Hospital, USA

## Background

The World Health Organization’s World Antimicrobial Awareness Week and the Centers for Disease Control and Prevention’s U.S. Antibiotic Awareness Week are observed annually from November 18 to 24.^1^ Both campaigns aim to raise awareness about the relationship between antimicrobial use and antimicrobial resistance. Participation typically includes public messaging through social media and educational outreach, as well as local activities within hospitals and clinics to engage healthcare professionals and patients in stewardship principles.^2^

Gamification of educational content has emerged as a strategy to increase participation and engagement during awareness campaigns such as Antibiotic Awareness Week.^3^ By incorporating puzzles, challenges, and interactive tasks linked to stewardship resources, gamified designs can transform passive information sharing into active learning experiences. The objective of this study is to evaluate the efficacy of a gamified digital intervention, 7 Days, 7 Quests, to increase engagement with antimicrobial stewardship resources during Antibiotic Awareness Week.

## Methods

This single‑center quality improvement project was conducted during Antibiotic Awareness Week, November 18 to 24, 2025, at a large academic medical center. Eligible participants were pharmacy and nursing staff. Antimicrobial Stewardship (AMS) program leaders and department managers distributed invitations by email on day 1 and day 5. Participation was voluntary, anonymity was allowed, and those who completed all seven quests could opt to enter a raffle for small stuffed microbe prize.

The intervention comprised seven daily quests delivered in Microsoft Forms posted on the AMS program SharePoint page (supplement). Each quest asked participants to use a specific antimicrobial stewardship resource to solve a puzzle or play a game, followed by an explanation of the puzzle/game content and an open-text feedback prompt. Learning objectives emphasized navigation to, and use of, local stewardship resources. Each quest was designed to be completed in under 10 minutes. The link to the AMS page was included in each quest. Participants needed to find the relevant document within the AMS page.

The primary outcome was engagement with the antimicrobial stewardship SharePoint page, measured by unique participants, completion of all seven quests, and page‑traffic volume. Secondary outcomes included time to complete quests, perceived clinical usefulness, and enjoyment of game formats. Rankings were combined with a Borda count, which assigns points to each rank and sums them to produce an overall order.^4^ Descriptive statistics summarized participation and traffic. The project used deidentified operational data, posed minimal risk, and received an IRB waiver.

## Results

**Figure 1.**
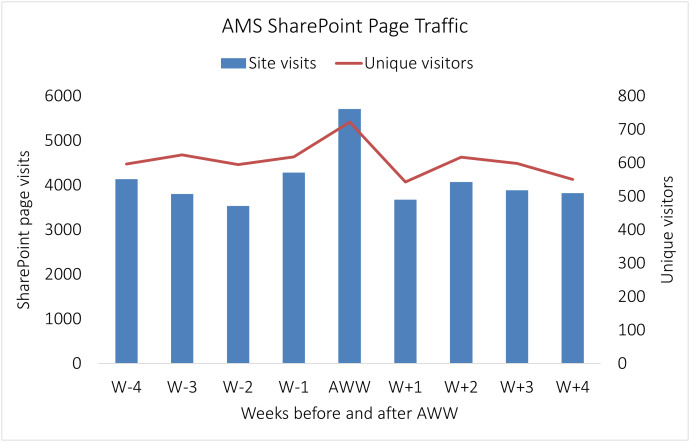
Weekly SharePoint page views and unique visitors four weeks before, during, and four weeks after Antibiotic Awareness Week. The primary *y*‑axis shows page views, the secondary *y*‑axis shows unique visitors, and the *x*‑axis shows time points from W − 4 through W + 4.

Seventy‑five unique participants engaged with the campaign, and 24 (32%) completed all seven quests. Mean time to complete quests ranged from 1.5 minutes for Quest 1 to 18 minutes for Quest 2. Participation declined over the week, from 73 for Quest 1 to 31 for Quest 7. During Antibiotic Awareness Week, SharePoint page views increased by 45% compared with baseline, and unique visitors increased by 19%. In the four weeks after the campaign, traffic returned to baseline levels (Figure 1).

Twenty‑eight valid responses were analyzed using a Borda count, which assigned points to each ranked item and summed them to generate consensus orders. For perceived clinical usefulness, the resulting order (highest to lowest total points) was blood culture identification (142), antibiogram interpretation (131), beta‑lactam allergy guidance (119), pneumonia treatment pathway (115), and antimicrobial dosing recommendations (113). For enjoyment of puzzle or game formats, the consensus order was rebus/pictogram puzzles (127), connections (115), acrostic (109), decision tree (97), and number sequence (79).

## Discussion

A gamified intervention was associated with a short-term increase in engagement with local antimicrobial stewardship resources. Directing clinicians to specific, locally endorsed materials through simple, interactive tasks appears to improve resource visibility during a defined campaign.

Gamified educational strategies have shown gains in engagement and motivation in healthcare education. A meta-analysis found that gamification improved retention of antimicrobial resistance content compared with conventional lectures.^5^ A recent prospective study of clinicians and students demonstrated that a lecture followed by interactive group games resulted in an improvement in pre-post assessment scores in distinguishing bacterial and viral infections (48% vs 94%), selecting appropriate empiric therapy (34% to 84%), and knowledge of resistant gram-negative bacteria (32% vs 78%).^6^

While these results are promising, most evaluations of game-based learning have focused on students rather than practicing clinicians. Undergraduate and postgraduate learners often participate in structured curriculum where games can be integrated into classroom or simulation settings, allowing for easier implementation and assessment. In contrast, clinicians in active practice face competing priorities, variable schedules, and limited protected time for education, which may influence both uptake and effectiveness of gamified interventions. A review of 53 gamification studies revealed that less than 20% of studies occurred in clinical or hospital-based environments involving practicing professionals.^7^ There is a paucity of data on whether similar strategies translate to sustained behavior change or improved clinical decision-making among experienced providers.

Limitations of the 7 Days, 7 Quests intervention include a single‑center setting limiting generalizability. Participation was voluntary, and a prize raffle may have introduced selection bias. Engagement was measured by page traffic and quest completion, not by knowledge retention, prescribing behavior, or clinical outcomes. Rankings of usefulness and enjoyment were self‑reported and may be influenced by response bias. Responders were all hospital employees. Physician and advanced-practice provider participation was limited by difficult consultant access to the hospital SharePoint site and restricted email outreach. Future interventions will assess if consistent integration of gamified tasks or quests results in sustained resource use, change in attitudes toward stewardship teams, and/or downstream prescribing behaviors.

## Supporting information

10.1017/ash.2026.10320.sm001 Jeffres et al. supplementary material Jeffres et al. supplementary material

## Supplementary material

The supplementary material for this article can be found at https://doi.org/10.1017/ash.2026.10320.
