# Supplementary material for: Seven days, seven quests: gamification to enhance engagement with antimicrobial stewardship resources during Antibiotic Awareness Week
Source: Antimicrob Steward Healthc Epidemiol. 2026 Mar 31;6(1):e72. doi: 10.1017/ash.2026.10320 (PMC13104535; doi:10.1017/ash.2026.10320)

Supplement

This document contains the screen shots of the puzzles and games used for the 2025 Antibiotic Awareness campaign called Seven Days, Seven Quests. Puzzles and games were built within Microsoft Forms to facilitate access and avoid firewalls for hospital employees.

Day 1 – Pictogram or rebus puzzle


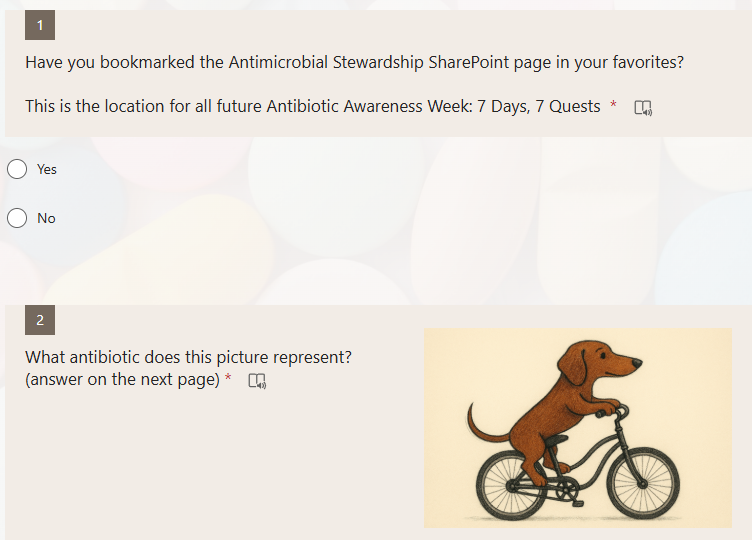


Day 2 - Pictogram or rebus puzzle


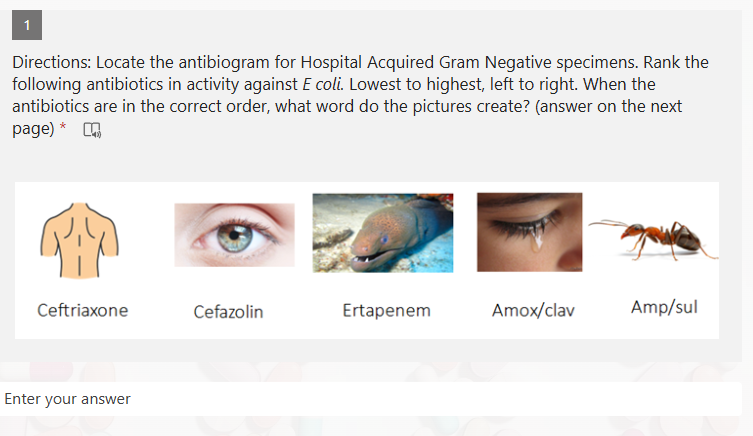


Day 3 – Acrostic


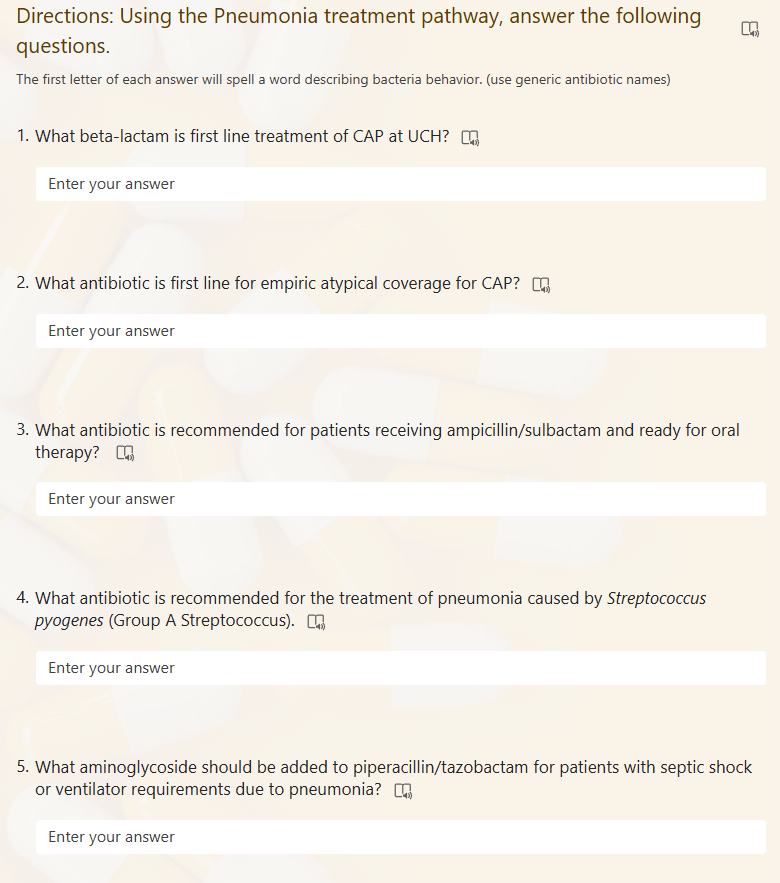


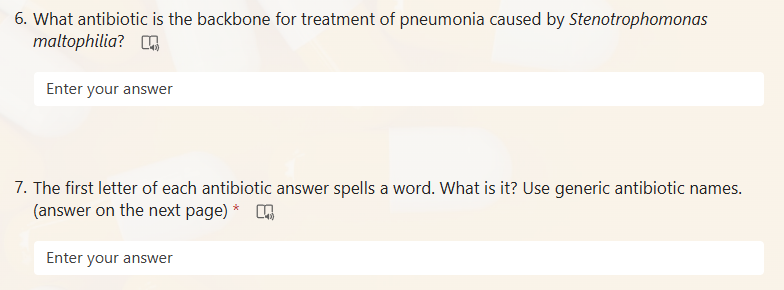


Day 4 – Connections or Connect 4


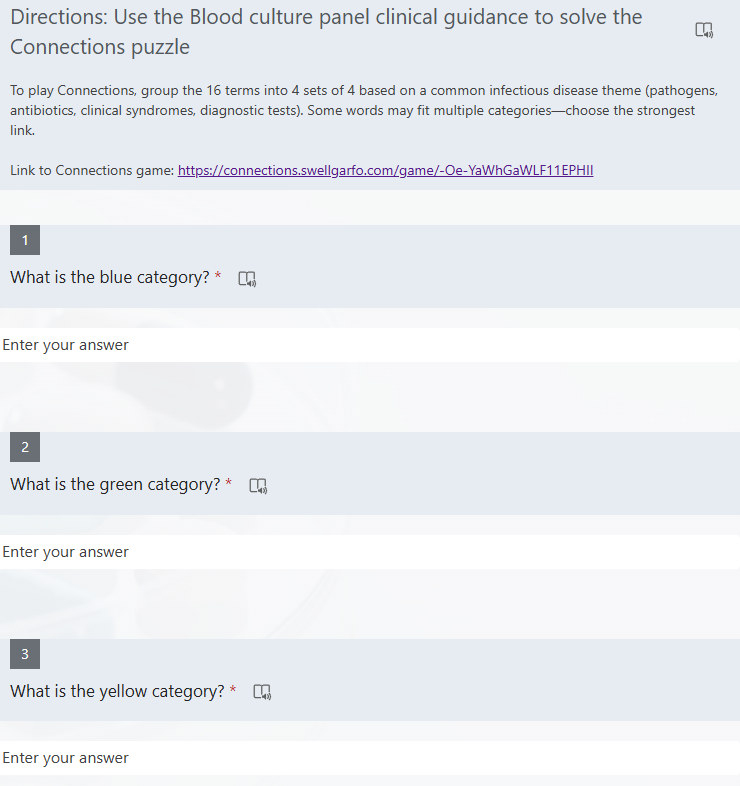


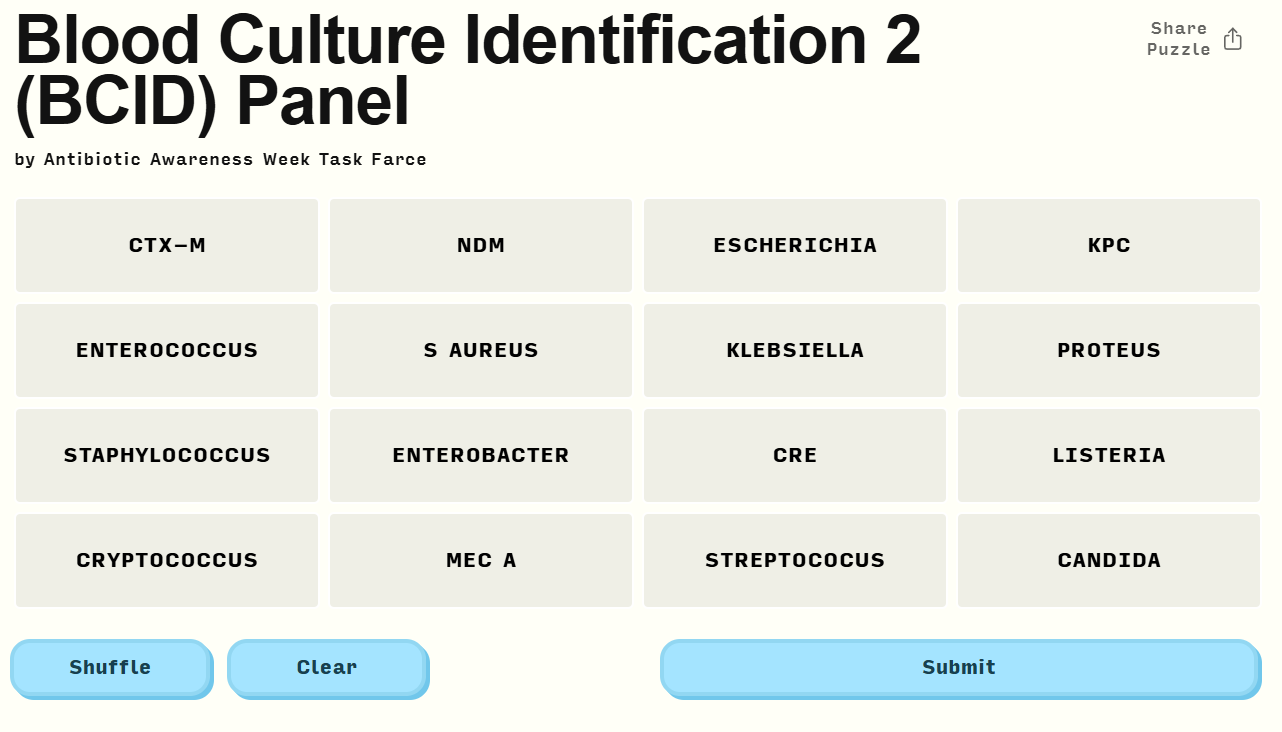


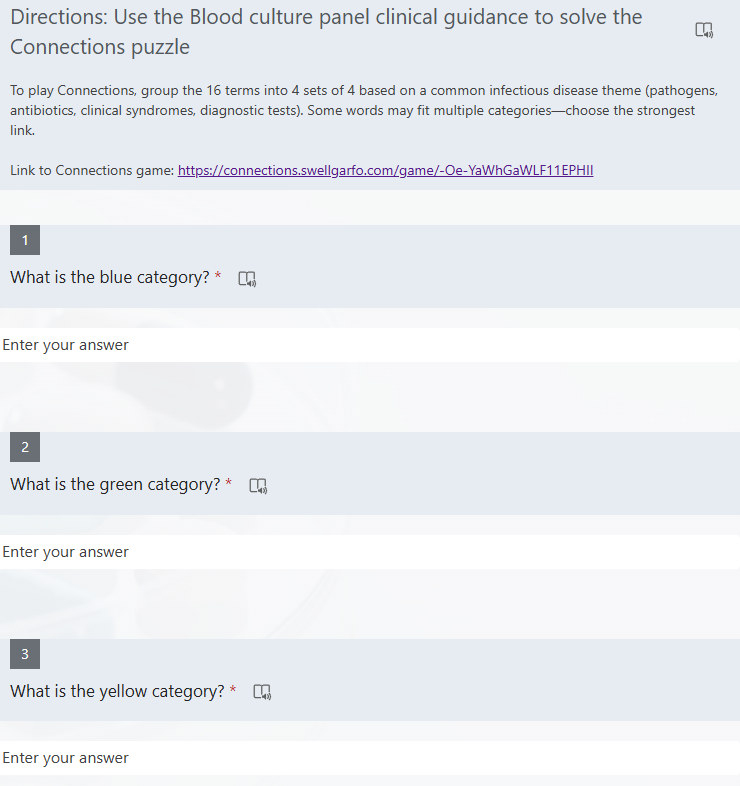


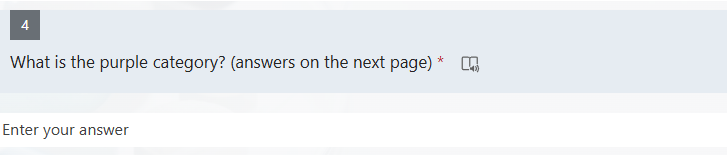


Day 5 – Number sequence


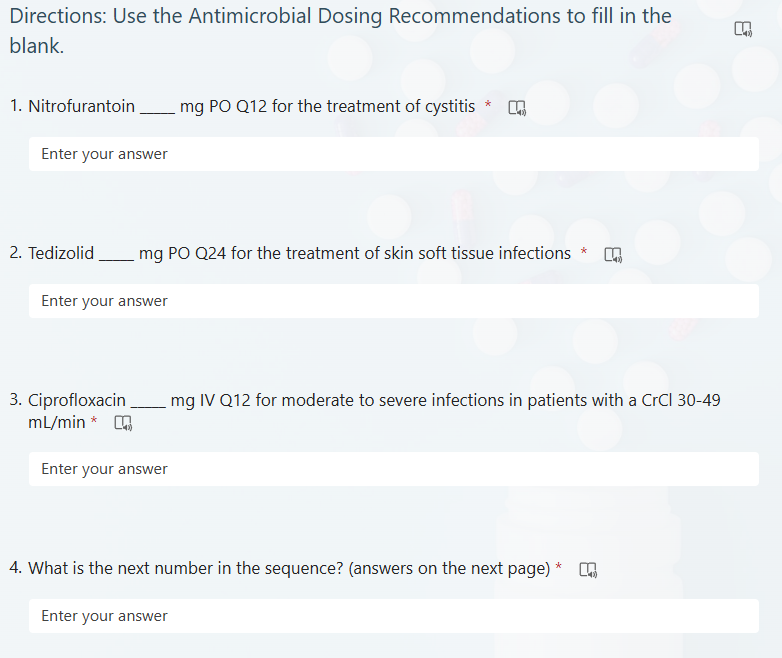


Day 6 – Decision tree pathway


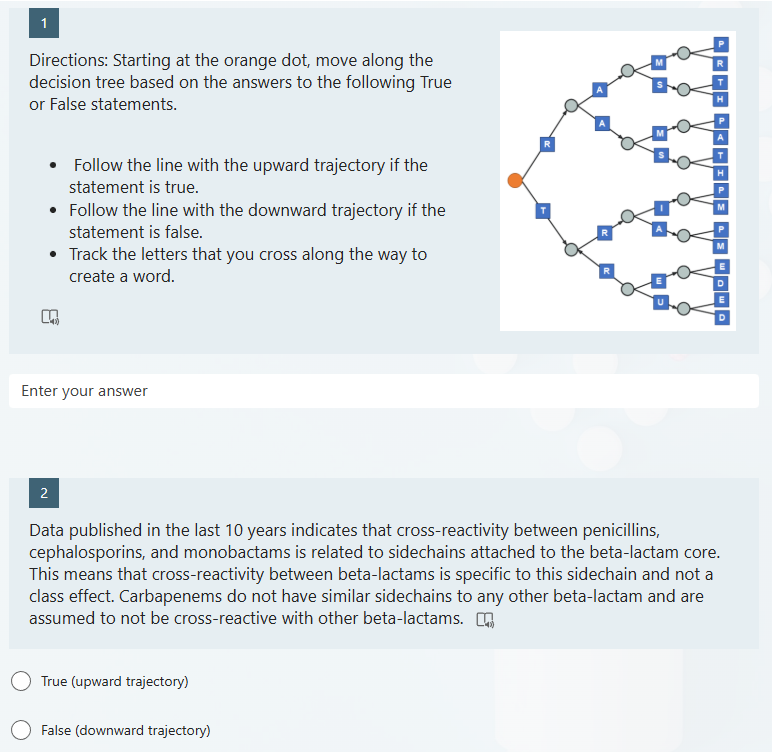


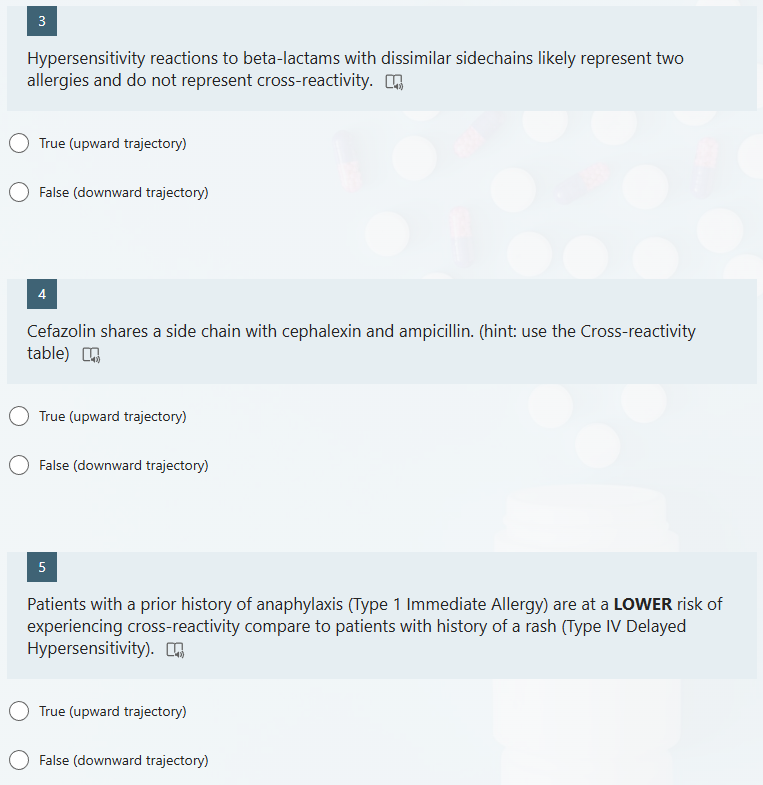


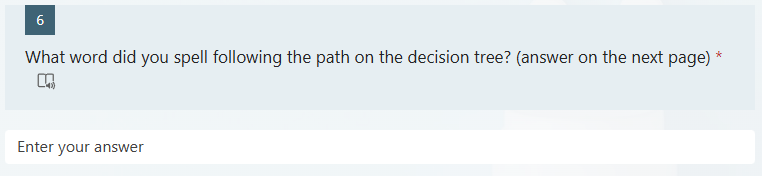


Day 7 – Multiple choice questions


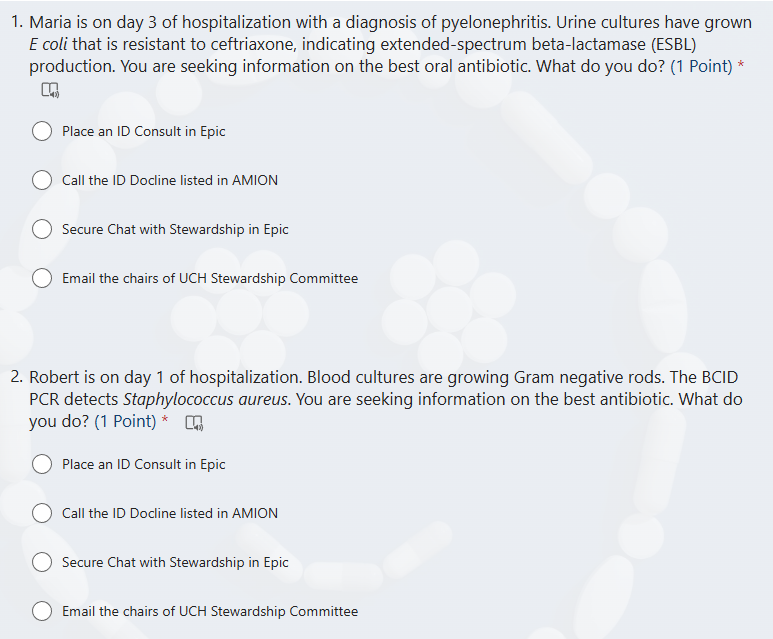


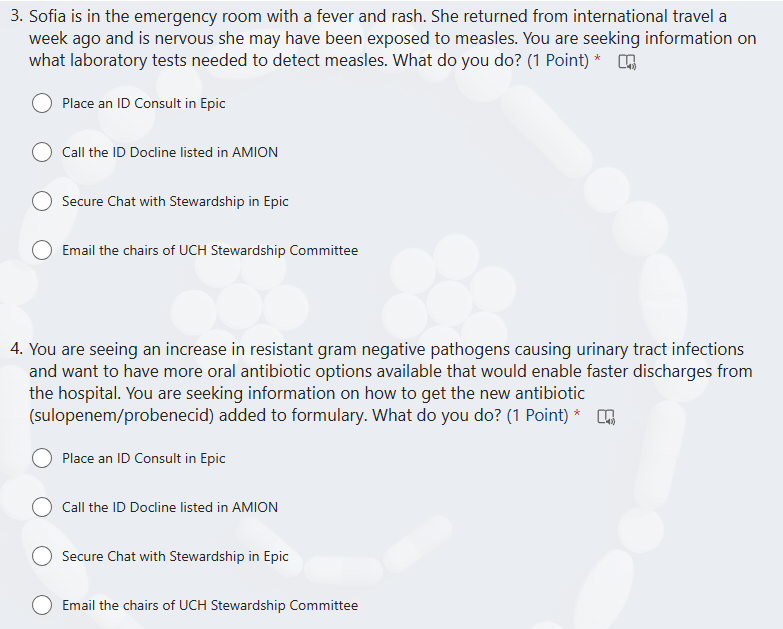


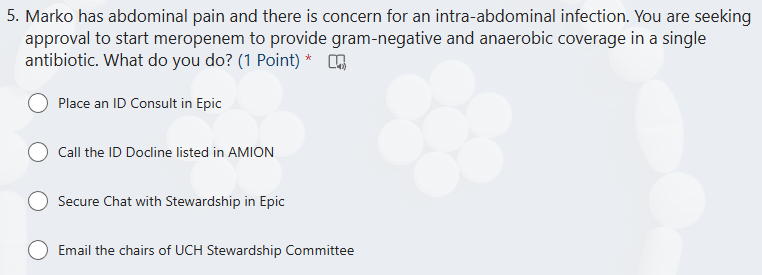

Supplement: Jeffres et al. supplementary material [file S2732494X26103209sup001.docx]
